# Crystal Chemistry and Design Principles of Altermagnets

**DOI:** 10.1021/acsorginorgau.4c00064

**Published:** 2024-10-23

**Authors:** Chao-Chun Wei, Erick Lawrence, Alyssa Tran, Huiwen Ji

**Affiliations:** † Department of Materials Science & Engineering, University of Utah, Salt Lake City, Utah 84112, United States; ‡ Materials Department and Materials Research Laboratory, University of California Santa Barbara, Santa Barbara, California 93106, United States; § Department of Chemical Engineering, California State Polytechnic University, Pomona, California 91768, United States

**Keywords:** altermagnet, crystal chemistry, crystal symmetry, magnetic materials, magnetic structure, materials design, anomalous Hall effect, spintronics, spin polarization

## Abstract

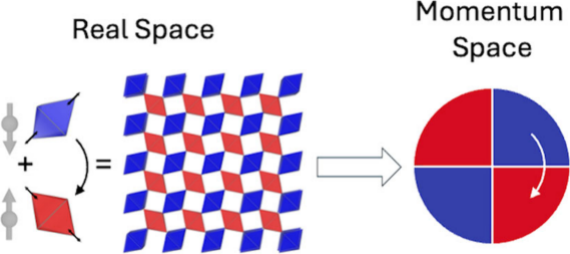

Altermagnetism was very recently identified as a new type of magnetic phase beyond the conventional dichotomy of ferromagnetism (FM) and antiferromagnetism (AFM). Its globally compensated magnetization and directional spin polarization promise new properties such as spin-polarized conductivity, spin-transfer torque, anomalous Hall effect, tunneling, and giant magnetoresistance that are highly useful for the next-generation memory devices, magnetic detectors, and energy conversion. Though this area has been historically led by the thin-film community, the identification of altermagnetism ultimately relies on precise magnetic structure determination, which can be most efficiently done in bulk materials. Our review, written from a materials chemistry perspective, intends to encourage materials and solid-state chemists to make contributions to this emerging topic through new materials discovery by leveraging neutron diffraction to determine the magnetic structures as well as bulk crystal growth for exploring exotic properties. We first review the symmetric classification for the identification of altermagnets with a summary of chemical principles and design rules, followed by a discussion of the unique physical properties in relation to crystal and magnetic structural symmetry. Several major families of compounds in which altermagnets have been identified are then reviewed. We conclude by giving an outlook for future directions.

## 1 Introduction

Magnetism is one of the most fundamental phenomena in materials with a long history of cognitive exploration and practical application. Ferromagnets (FMs) were first utilized by humans due to the nonzero overall magnetization. They feature a broken time-reversal symmetry (TRS) at all *k* points in the electronic structure with nonrelativistic spin splitting into the so-called majority and minority bands, leading to effects such as spin-transfer torque,^1^ giant or tunneling magnetoresistance^2^ useful for making memory devices or spin valves. In the context of magnetic order, breaking TRS means , wherein  and ↑ are momentum and spin, respectively. Antiferromagnets (AFMs), on the other hand, remained unrecognized until 1933 due to their vanishing magnetization originated from a magnetic structure wherein neighboring spins pointing in exactly compensating directions.^3^ Because AFMs do not generate stray fields, they can be more tightly packed in devices yet are still free from cross interference and are robust against external perturbations. For this reason, AFM materials traditionally found use as inactive pinning layers in spintronic devices for their insusceptibility to external magnetic fields.^4^ Recently, significant interest lies in developing spintronic memory devices using AFMs as active components to achieve higher energy efficiency than electronics and orders of magnitude faster spin dynamics than their FM counterparts.^5^

To realize electrical write-in and read-out in AFM memory devices, the magnetic order and hence the anisotropic magnetoresistance tensor must be susceptible to an electrical current. Specifically, a current first needs to be polarized and then transfers its polarization to the magnetic order in the AFM to modify the resistivity tensor for electrical read-out. Several mechanisms have been established in emerging AFM material platforms to realize such functionality. The types of materials can be briefly summarized below.

**Figure 1:**
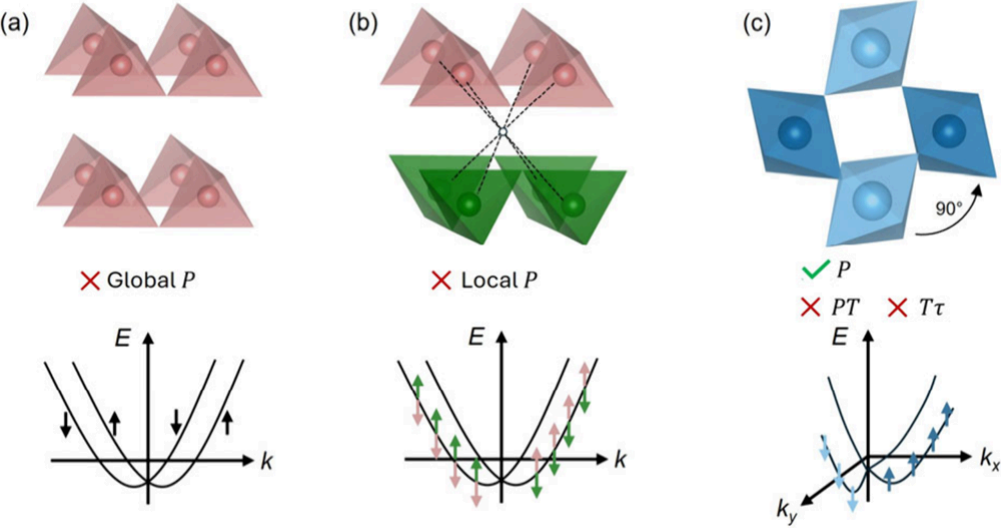
Three mechanisms for realizing spin polarization in AFMs. (a) Non-centrosymmetric (lacking global inversion (or parity *P*)) crystal structure leads to the Rashba or Dresselhaus effect. (b) An inversion center connects local non-centrosymmetric units (lacking local parity *P*), causing real-space separated spin polarization. (c) Two opposite-spin sublattices connected by rotation only (no *PT* or *Tτ*, τ is translation, *T* is time-reversal symmetry), leading to *k*-dependent spin splitting texture with the same rotation symmetry.

- Materials lacking global inversion symmetry. An example would be Fe_1/3_NbS_2_,^6^ which has a non-centrosymmetric space group of *P*6_3_22, resulting in a spin–orbit coupling (SOC) induced Rashba or Dresselhaus effect^7^ and spin polarization (Figure 1a). The same phenomenon can also show up in heterostructures due to interfacial asymmetry.
- Materials lacking local inversion symmetry. This idea was first demonstrated in 2016 based on CuMnAs^8^ and then on Mn_2_Au.^9,10^ In these materials, the local coordination environments around the magnetic centers, i.e., Mn, are square-pyramidal and lack inversion symmetry although the entire crystal structure preserves the inversion symmetry.^11^ Meanwhile, the two opposite-spin sublattices are interchangeable by the combination of TRS and inversion (*PT*). This causes the Rashba/Dresselhaus effect to rise locally, inducing real-space spin polarization that is spatially separated in a staggered, albeit globally compensated manner (Figure 1b).

Note that both Mechanisms (i) and (ii) rely on relativistic quantum mechanics, that is the Rashba spin–orbit interaction to polarize an originally nonpolarized electrical current, which then strongly couples with the two AFM sublattices. The spin polarization *p* induced by a current is given by
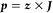
where ***z*** is a unit vector along the non-centrosymmetric polar direction and ***J*** is the applied current.^9^ Note that because the two Mn sublattices in Mechanism (ii) have their local environment inverted, their local polarizations on the current are also opposite. The polarized currents then exert a torque of certain origins to rotate the local magnetic moments and the resistivity tensor by SOC to enable electrical read-out. The effects are relativistic in nature and are typically very weak. As a result, materials exploration is limited to those containing high-*Z* elements with strong SOC. Furthermore, a large write-in current density is still needed for these materials, on the order of >10^6^ A/cm^2^,^6,13^ which compromises the low-energy advantage of spintronics.

**Figure 2:**
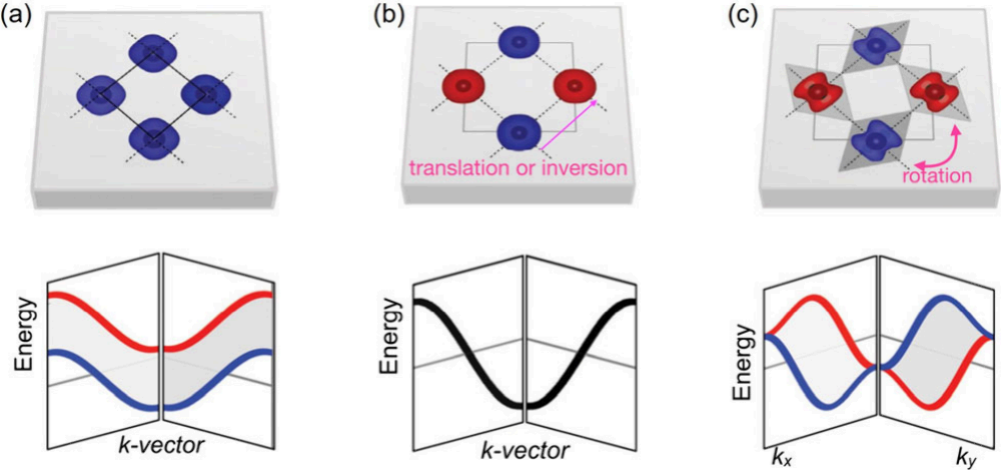
Real-space magnetic orders (top) and their corresponding momentum-space electronic structures (bottom). (a) Collinear FM order with non-*k*-dependent spin splitting (red and blue for minority and majority spin channels). (b) Conventional AFM of translation or inversion-related spin sublattices shows spin-degenerate bands. (c) Altermagnetic order with rotation-related spin sublattices and *k*-dependent band splitting. Reproduced from ref (12). Available under a CC-BY 4.0 license. Copyright 2022 Libor Šmejkal.

- Crucially, a third type of materials that allows current polarization as well as an electrical readout of the magnetic order are altermagnets (a term very recently coined by Jungwirth and colleagues). Altermagnets represent a new type of magnetic phases beyond the conventional dichotomy of FM and AFM,^12,14^ whose discovery was mainly a result of the recent search for AFM materials platforms for spintronic devices.^15^ Altermagnetic materials possess globally compensated magnetization like an AFM and directional spin polarization like an FM at the same time.^16^ Its uniqueness is rigorously proven by symmetry classification.^17^ The two opposite-spin sublattices of the magnetic structure are connected by the combination of rotation symmetry and TRS^12^ (Figure 1c). The rotation can be proper or improper and symmorphic or nonsymmorphic. The sublattices cannot be connected by translation or inversion symmetry, of which the former incurs a Type IV magnetic space group as defined by Bradley and Cracknell while the latter constitutes the second mechanism mentioned above.^18^ The resulting altermagnets exhibit a polarized spin-momentum locking in the reciprocal space with a *k*-dependent sign, i.e., the spin-up bands are lower in energy along certain *k* paths but are higher in energy along separate, rotation-connected *k* paths, with the same symmetry as in real-space sublattices. The spin texture is shown in Figure 2c, in contrast to that of FM or conventional AFM (Figure 2a,b). More importantly, the resultant spin-splitting does not originate from relativistic effects and can still be significant in lighter and more earth-abundant compositions based on e.g. Fe and Mn. Besides, broken centro-symmetry locally or globally is unnecessary, which greatly expands the material candidate pool. Examples span insulating CuF_2_,^14^ MnF_2_,^18^ MnTe,^14^ and metallic RuO_2_,^19^ Mn_5_Si_3_, and CrSb.^12^ Altermagnetic materials are predicted, depending on the specific symmetries exhibited, to host macroscopic responses such as spin-polarized conductivity, spin-transfer torque, anomalous Hall effect (AHE), tunneling magnetoresistance, and giant magnetoresistance^12,20,21^ that are highly useful for electrical write-in and read-out operations in AFM memory devices.

Despite the predicted universality and excellent spintronic functionality of altermagnetism, material realizations and measurements are still scarce due to several bottlenecks. Spintronics has been a field historically dominated by the thin-film community for it is the needed technology for industrialization.^22^ But thin-film methods cannot explore many materials or freely test the anisotropy because it requires careful selection of a compatible substrate for each material grown along a certain direction. In the meantime, the identification of an altermagnetic phase and its spin group analysis must be done based on a known magnetic structure. Currently, neutron diffraction is the dominant method for new magnetic structure determination but requires bulk materials due to the weak interaction of neutrons with matter. Recognizing these hurdles, materials and solid-state chemists can make unique contributions to this emerging field through new materials discovery by leveraging neutron diffraction to determine the magnetic structures and analyze spin groups as well as bulk crystal growth for the exploration of exotic properties.

In this article, we first review the established symmetry classification based on spin groups for the identification of altermagnets, followed by a summary of chemical principles and design rules for obtaining the desired crystal symmetry and band splitting, as well as the extended concept of supercell altermagnets. We move on to talk about the unique physical properties of altermagnets in relation to crystal and magnetic structural symmetry. Several major families of compounds in which altermagnets have been identified and measured are then reviewed, including the rutile, NiAs, Mn_5_Si_3_ types, and magnetically intercalated Nb/Ta dichalcogenides. We conclude by giving an outlook for future directions from the perspective of materials chemistry.

## 2 Symmetry Principles and Design Rules

### 2.1 Unique Properties Dictated by Spin Groups

The discovery of new phases of matter and the prediction of emerging properties have been increasingly enabled by the rediscovery and reclassification of crystalline symmetries. Systematic classification of spin symmetries in decoupled spin and crystal space showed that altermagnetism can arise in 6 of the crystal systems (excluding triclinic). Three symmetry groups were defined following the convention of theoretical works,^17^ i.e., G, A, and H, wherein G is the crystalline point group, H is the same-spin sublattice point group, and A contains symmetry transformation(s) that connect the atoms in the two opposite-spin sublattices (summarized in Table 1, reproduced from ref (14), available under a CC-BY 4.0 license. Copyright 2022 Libor Šmejkal). Note that group A should only contain rotation symmetries (improper/proper, symmorphic/nonsymmorphic) but not translation or inversion to be qualified as altermagnetic. This is because, as the two key criteria for altermagnet identification in collinear cases, the two opposite-spin sublattices should not be connected by centro-symmetry (inversion) or translation symmetry. In most common cases, the second criterion implies the necessity of a zero magnetic propagation vector ***k*** of the magnetic structure although there could be exceptions as discussed below in Section 2.5. Also, depending on whether H contains a mirror perpendicular to the highest-order rotation axis (i.e., /*m*), the spin-momentum locking in reciprocal space can be either planar (P) or bulk (B), respectively. In addition, as the group H contains a higher-order rotation symmetry (or multiple rotations), there are increasingly more spin-degenerate nodal planes in the momentum space without considering SOC, i.e., 2, 4, or 6, referred to as *d*-, *g*-, and *i*-wave types, respectively.^14^ As a result, six spin-momentum locking types are classified in Table 1 and are viewed down the rotation axis. The red and blue color blocks represent opposite spins in the momentum space and the white gaps in between are spin-degenerate nodal planes parallel to the rotation axis. Note that the planar types (P-2, P-4, P-6) only have nodal planes parallel to the rotation axis, while the bulk types (B-2, B-4, B-6) have an additional nodal plane perpendicular to the rotation axis that is not visible in the 2D views in Table 1. Theory predicts that all six types give rise to anomalous Hall effect (AHE) and tunneling magnetoresistance. However, only the *d*-wave types (P-2 and B-2) lead to spin-polarized conductivity, as a result of the Fermi surface anisotropy when biased in orthogonal directions and giant magnetoresistance that are useful for electrical write-in and read-out.^12^ This implies that for the most versatile spintronic memory applications, the ideal altermagnets should not contain high-order (3-, 4-, or 6-fold) rotation symmetries in the same-spin sublattice H.

**Table 1:**
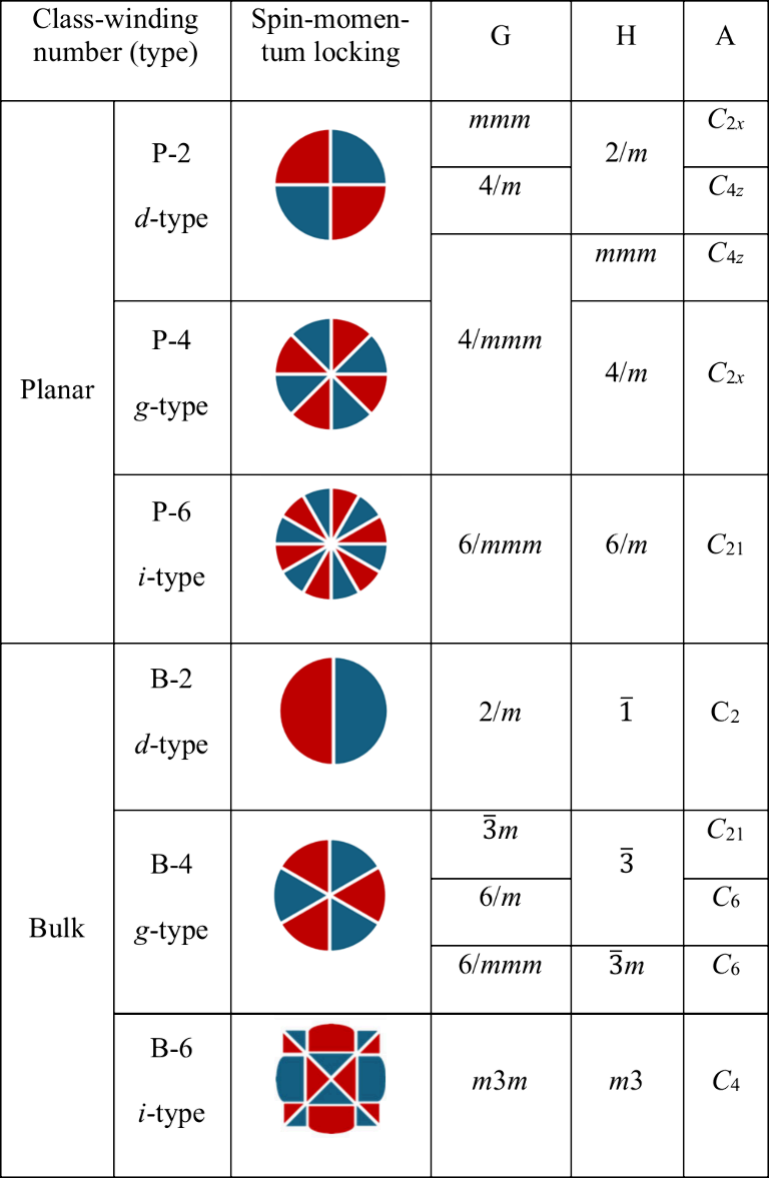
Classification of Altermagnetic Spin-Momentum Locking

### 2.2 Chemical Principles for Lowering Same-Spin Sublattice Symmetry

**Figure 3:**
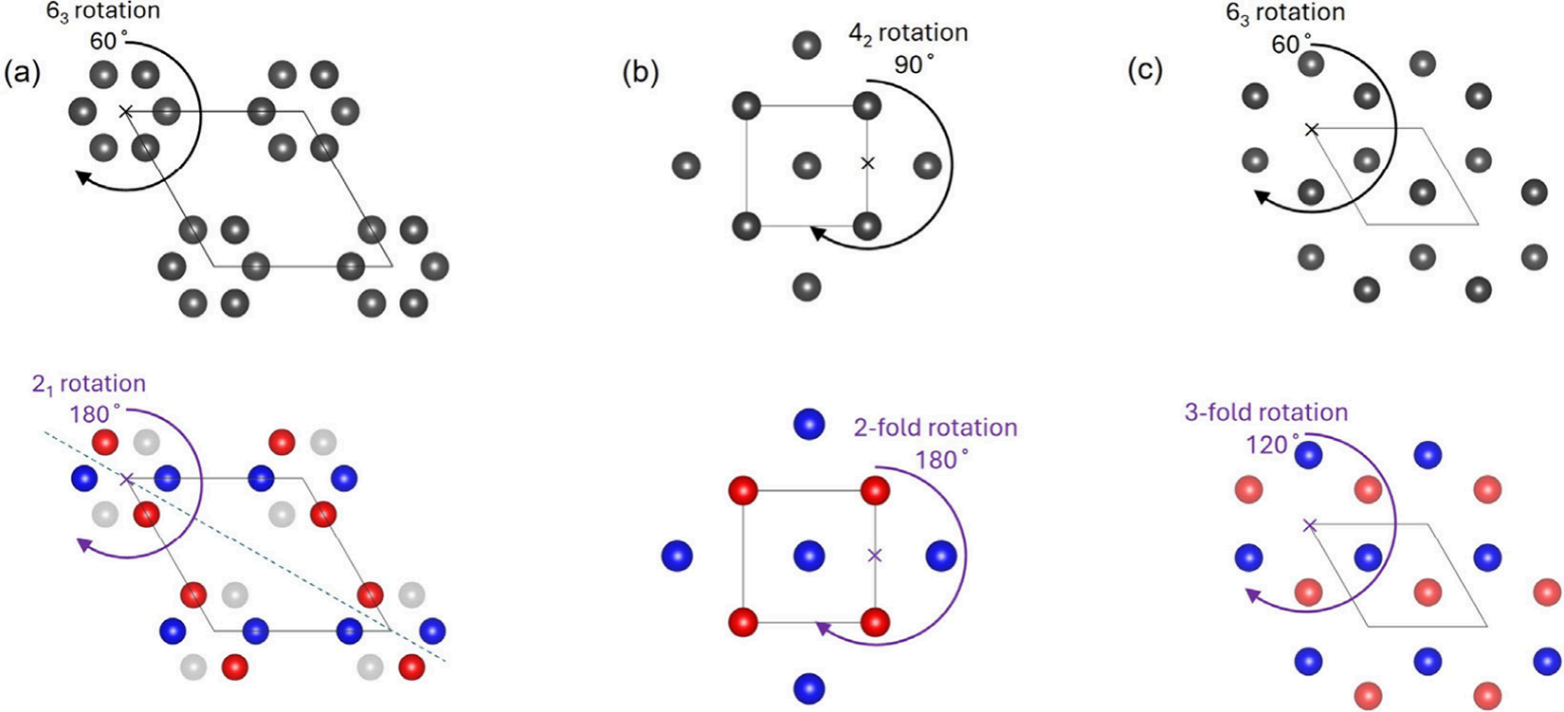
Crystal symmetry before (upper) and after AFM transition (bottom). Nonmagnetic atoms are omitted for easier visualization. Red and blue atoms are of opposite spins. (a) Schematic of Mn atoms in the Wyckoff position in Mn_5_Si_3_ with a 6_3_ rotation symmetry between equivalent atoms. The lattice symmetry reduces to orthorhombic after 2/3 of the sites turn magnetically ordered. 2_1_ rotation remains in the same-spin sublattice in the AFM state. (b) Cation sites in the rutile structure with 4_2_ rotation between equivalent atoms. 2-fold rotation is retained in the same-spin sublattice in the AFM state (not 4-fold because of O coordination that is not shown). (c) Intercalant sites in the VNb_3_S_6_ structure with 6_3_ rotation to connect equivalent atoms. In the AFM state, because of the FM order in the same layer (blue and red atoms are in different layers), a high-order 3-fold rotation is kept in the same-spin sublattice.

To facilitate a spin-momentum locking with only two nodal planes (i.e., B-2 and P-2) and the subsequent rise of spin-polarized conductivity as well as giant magnetoresistance for spintronic memory, a same-spin sublattice H of no high-order rotation symmetry is favored. Besides choosing candidates from monoclinic/orthorhombic crystal systems, another symmetry-lowering strategy is to find materials in which not all magnetic atoms develop a magnetic order. For example, Mn_5_Si_3_, a predicted altermagnet,^23^ has 6-fold screw rotation in a paramagnetic state. Upon AFM transition, only 2/3 of the Mn atoms in the Wyckoff position that are otherwise connected by 6_3_ screw rotation are magnetically ordered.^24,25^ The magnetic structure thus loses its high-order rotation and lowers its symmetry to orthorhombic and hosts *d*-wave spin texture.^24^ The Mn sites that turn partially magnetic and AFM ordered are depicted in Figure 3a. Before the AFM transition, there is high-order 6_3_ rotation connecting the equivalent Mn atoms (Figure 3a, upper). Once in the AFM state, 2/3 of the Mn carry ordered moments and low-order 2_1_ rotation is retained in a same-spin sublattice (Figure 3a, bottom). Nevertheless, materials with only a portion of its magnetic atoms carrying ordered moments are extremely rare.

As a more universal design strategy, having opposite moments in a same atomic layer that is perpendicular to the high-order rotation axis can effectively lift the high-order rotation, because magnetic atoms that are otherwise connected by high-order rotation are then divided into separate sublattices. In other words, materials exhibiting in-plane AFM order are particularly desirable for realizing *d*-wave altermagnetism and current polarization if electrically conducting. For example, the rutile structure, which features several predicted altermagnets including RuO_2_, MnF_2_, NiF_2_, and CoF_2_, contains 4-fold screw rotation along *c* in its nuclear crystal structure (Figure 3b, upper). Upon the AFM transition, the magnetic moments adopt an in-plane collinear AFM order, thus removing the 4-fold rotation in each same-spin sublattice H^26^ (Figure 3b, bottom). The resulting spin-momentum symmetry is of the P-2 type with two nodal planes and can support spin-polarized conductivity if electrically conducting. On the contrary, V_1/3_NbS_2_ features in-plane moments with FM interaction within the layer and AFM interaction across layers.^27^ As a result, each same-spin sublattice retains 3-fold rotation with 2_1_ rotation between the opposite-spin sublattices (Figure 3c, the blue V atoms are in a layer above the opposite-spin red V atoms), leading to B-4 type spin-momentum locking. However, it should also be noted that certain in-plane AFM order can lead to a nonzero magnetic ***k*** vector and a supercell. The resulted combination of translation and TRS will, in most cases, render the material nonaltermagnetic.

Controlling the type of AFM magnetic order, however, is highly nontrivial. Depending on the coupling mechanism between magnetic moments, different design strategies may be utilized to induce the desired in-plane AFM. If the Ruderman-Kittle-Kasuya-Yosida (RKKY) interaction dominates, e.g., in magnetically intercalated layered transition metal chalcogenides such as Co_1/3_NbS_2_ and V_1/3_NbS_2_, the metal–metal distance is an effective tuning handle for achieving a desirable sign in the interaction.^28,29^ Alternatively, if superexchange interactions dominate, e.g., in oxides or fluorides, then the metal–ligand–metal angle, electron configuration, and electronegativity of the ligand or the metal are the relevant parameters for modifying the interaction type.^30^

### 2.3 Design Rules for Enhancing Spin Splitting

**Figure 4:**
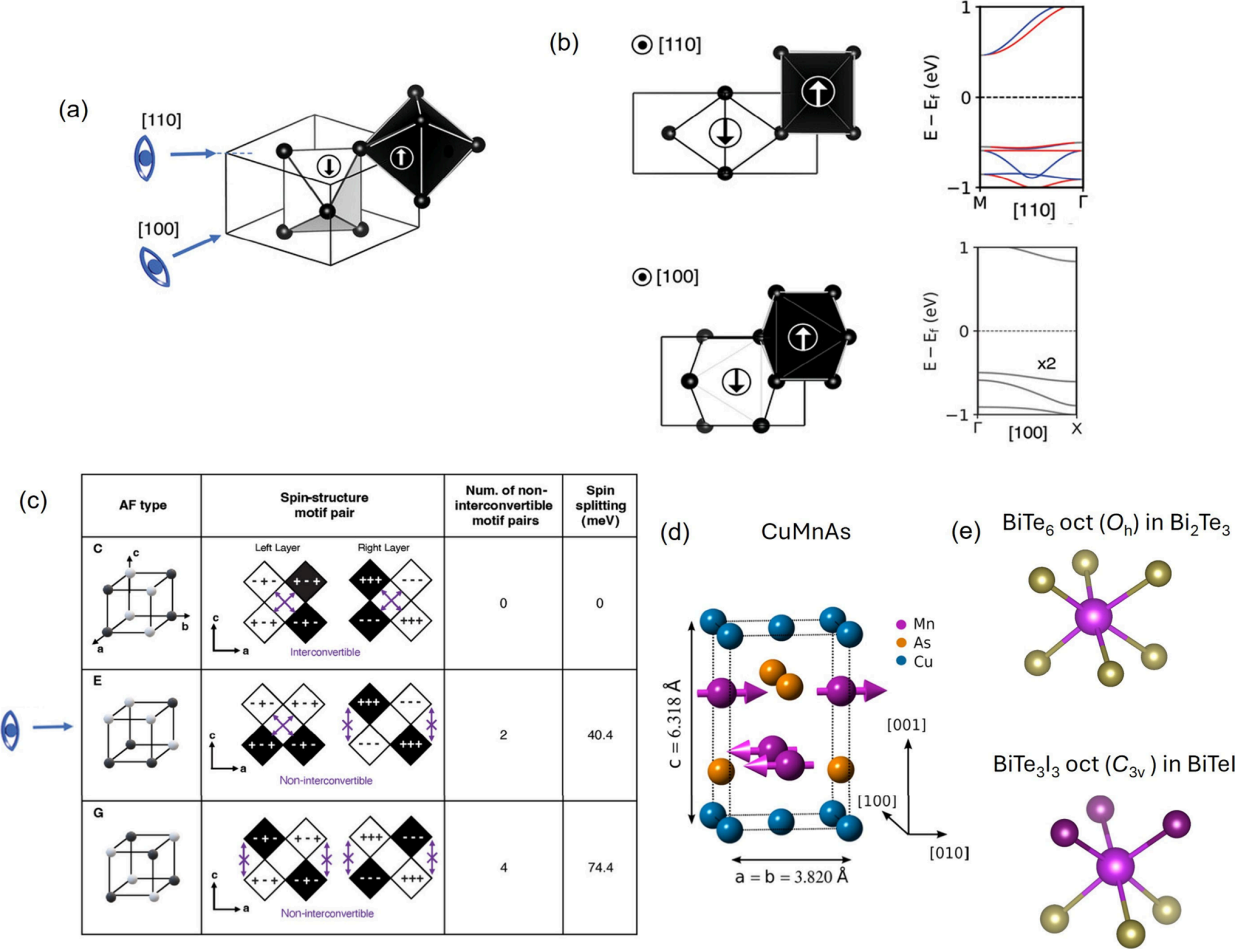
(a) Schematic of an intuitive way of estimating the magnitude and ***k*** direction of spin splitting. (b) Contrast between views down different crystal axes and the corresponding band splitting/degeneracy. (c) Octahedral tilting in *a*^–^*b*^+^*a*– tilted LaMnO_3_ and their interconvertibility between opposite-spin motifs. “+” and “–” indicate in-phase and out-of-phase following Glazer’s convention. (d) Crystal structure of thin-film stabilized tetragonal CuMnAs. (e) Octahedra coordination in BiTeI and Bi_2_Te_3_ with and without mixed-anion substitution. (a-c) Reproduced with permission from ref (31). Copyright 2023 John Wiley and Sons. (d) Reproduced from ref (43). Available under a CC-BY 4.0 license. Copyright 2015 P. Wadley.

Enhanced spin splitting/polarization is favored for more pronounced spintronic properties. Intuitively, magnetic atoms are embedded in certain local coordination environments made of nonmagnetic atoms, i.e., local motifs. The spin polarization in altermagnets is induced by crystal-field anisotropy created by these local motifs. Between the local motifs of the opposite-spin sublattices, the more mutually tilted the local anisotropy axes are, the larger the spin splitting is, as pointed out by Zunger et al.^31^ These authors summarized straightforward empirical rules for solid-state chemists to qualitatively estimate the magnitude and ***k*** direction of spin splitting based on the mutual tilting of local motifs.^31^ For example, in the prototypical altermagnetic system of RuO_2_ with a rutile-type structure (Figure 1c), the magnetic Ru atoms are in octahedral local motifs which are not perfectly symmetric. Instead, the apical O anions are displaced closer to the metal center which enhances anisotropy. Between the two opposite-spin sublattices, the anisotropy axes are mutually rotated by 90° in the *ab* plane. Visual inspection of the crystal structure along different directions yields the following insight (Figure 4a,b): the local motifs of the two opposite-spin sublattices appear the most different if viewed along the face-diagonal [110] and accordingly the electronic bands experience the biggest spin splitting along Γ – *M*. If viewed along [100], the octahedral motifs look identical and accordingly the bands are spin-degenerate along Γ – *X*, which is also the nodal-plane direction. AFM insulators of the same rutile structure, NiF_2_ and MnF_2_, also exhibit most pronounced spin polarization along Γ – *M*.

The magnitude of spin splitting also depends on the type of AFM order. For example, among the versatile manganate perovskites, LaMnO_3_ has its La ions (or other rare-earth elements) too small to fit without distortion into the cuboctahedral voids, leading to collective tilting in the MnO_6_ octahedral network to lower the global symmetry. The octahedral tilting might lead to spin splitting but the magnitude of splitting still depends on the specific AFM type. With a same *a*^–^*b*^+^*a*– tilting pattern, a C-type AFM would cause the spin splitting to vanish while E-, or G-types have increasing nonzero spin splitting as the symmetry further lowers.^31^ As shown in Figure 4c, when viewed along ***b***, the opposite-spin environments become more dislike (aka., noninterconvertible) from C-, E-, to G-type AFM. Accordingly, the band splitting along the direction increases. In the meantime, the band splitting becomes more significant when the overall octahedral tilting angles increase. Given the intricate interplay between spin and orbital degrees of freedom in perovskites,^32,33^ the magnetic phases in this example might be tunable via chemical substitution in the rare-earth site.

Finally, mixed anions have long been applied as a strategy in designing nonlinear optics^32,34^ with second-harmonic generation properties. A mixed-anion coordination motif may break centro-symmetry locally like in the case of thin-film stabilized tetragonal CuMnAs.^35^ Mn is surrounded by Cu and As, forming a halved cuboctahedral environment, as shown in Figure 4d. Even global centro-symmetry can be broken if no inversion centers would reconnect the local motifs,^36,37^ like in the comparison between Bi_2_Te_3_ and BiTeI (Figure 4e). An BiTe_6_ octahedron in Bi_2_Te_3_ has high *O*_h_ symmetry.^38^ Its local inversion symmetry is lifted once half of the Te is replaced by I to form BiTe_3_I_3_ (*C*_3v_). The resulted compound BiTeI is non-centrosymmetric with giant Rashba-type spin splitting.^7,39^ For the design of altermagnetism, although breaking centro-symmetry is unnecessary,^18^ more than one type of anions (or more generally, coordinating ions) can still lower symmetry and enhance local anisotropy, potentially causing larger spin polarization when the anisotropy axes of motifs are mutually tilted between spin-sublattices. The synthesis of mixed-anion compounds is nontrivial and requires consideration of size, electronegativity, and valence.^40−42^ First, common anions such as chalcogens and halogens are low in valence and tend to favor 2D layered or 1D tunneled structures while pnictogens and Group 14 elements tend to form 3D networks. Besides, anions of large contrast in sizes or electronegativity, such as O and chalcogens or O and halides, despite being more prone to phase separation, would favor long-range anion order and enhanced collective anisotropy, while similar anions such as S and Se or Se and Te tend to form disorder. Finally, the soft–hard acid–base theory can be used to predict the structural stability if mixed cations and anions are present, i.e., soft cations (of large sizes and low charges) tend to be coordinated by soft anions.

### 2.4 Magneto-crystalline Anisotropy

Magneto-crystalline anisotropy is one type of magnetic anisotropy in which a crystalline material magnetizes more readily (i.e., saturates at a lower field) along a specific crystallographic direction, which is referred to as the “easy axis” for FMs, than the other. The primary source of magneto-crystalline anisotropy is SOC because the spins of electrons cannot freely align along the external field because of their coupling to the electron orbitals, which are coupled to the crystalline lattice.^44^

In AFMs, the orientation of moments, known as the “Néel vector” for collinear magnetic orders, has profound consequences in the properties of altermagnets.^45^ It determines the symmetry of the magnetic structure, the presence or absence of AHE, or the direction of spin polarization. The direction of the magnetic moments can be reoriented, depending on the magnitude of magneto-crystalline anisotropy, using an external magnetic field, optical means,^46^ a strain effect,^47^ an electric field,^48^ or chemical substitution.^49^

### 2.5 Supercell Altermagnets

Recently, the scope of altermagnets was further expanded to include magnetic phases with nonzero propagation vectors, i.e., ***k*** ≠ (0,0,0), termed supercell altermagnets.^50^ Oftentimes, a collinear AFM exhibiting the combination of *T* and translation symmetries falls in a type IV magnetic space group and is thus a trivial AFM. However, when a more complex magnetic order develops, in which the combination of *T* and translation symmetries is still lacking, altermagnetism arises if the combination of inversion and *T* is also absent. A survey in the MAGNDATA database identified four possible supercell altermagnets, i.e., MnSe_2_, CsCoCl_3_, RbCoBr_3_, and BaMnO_3_.^50^ For these candidates, the two opposite-spin sublattices are connected by combination of noninteger translation of the magnetic unit cell and mirror symmetries.

## 3 Crystal Symmetry and Emerging Properties

### 3.1 Anomalous Hall Effect

One predicted hallmark of altermagnets is the emergence of anomalous Hall effect (AHE).^12^ AHE was first discovered in FMs wherein the time-reversal symmetry is broken while in the presence of spin–orbit coupling to give rise to dissipation-less conductivity transverse to the direction of the electric field. AHE was later found in coplanar noncollinear AFMs and complex noncoplanar spin structures like skyrmion systems.^51−54^ Altermagnets can also host relativistic AHE like FMs with the help of SOC and in the absence of an inversion or crystal translation symmetry between the two opposite-spin sublattices, defying conventional wisdom that the AHE scales up with total magnetization. In all cases, the intrinsic topological Berry curvature is believed to be the main contributor and can be quantified theoretically.^20^ The difference, however, is while an FM enables a nonzero Hall vector regardless of the orientation of the magnetization ***M***, the Hall pseudovector **σ** in an altermagnet can be either allowed or forbidden depending on the symmetry as well as the relative orientations of the Néel vector ***L*** = ***m***_1_ – ***m***_2_ (***m***_1_, ***m***_2_ represent the antiparallel magnetic moments) and the rotation axes in crystal symmetries. The Hall pseudovector **σ** = (*σ*_*zy*_, *σ*_*xz*_, *σ*_*yx*_) is odd under time reversal and transforms like a magnetic dipole moment under crystal symmetry operations. A Hall conductivity transverse to the Hall vector and the electric field can be induced following .

The orientation of the Hall vector is further restricted by magnetic crystal symmetry and can be predicted.^20^ Based on a systematic classification, only 10 magnetic Laue groups are identified to host nonzero Hall vectors. Note that these Laue groups are obtained from magnetic point groups by converting any inversion or mirror symmetry to identity or 2-fold rotation, respectively. The lowest-symmetry Laue group contains only the identity symmetry. In this case, a nonzero Hall vector is allowed, though its orientation is not confined by symmetry but depends on other material properties. The next group 2′, i.e., combining a 2-fold rotation and spin inversion, dictates the Hall vector to be within a plane perpendicular to the rotation axis. For the eight other Laue groups, 2, 3, 4, 6, 2′2′2, 42′2′, 32′, and 62′2′, the nonzero Hall vector must be along the unprimed rotation axis. Such symmetry classification is highly useful when designing the orientation and geometry of a Hall bar to measure AHE. These identified magnetic Laue groups suggest that AHE can arise in all the six categories of spin-momentum types as summarized in Table 1.

Other than collinear altermagnets with only two magnetic atoms per unit cell, where the presence and orientation of the Hall vector also depend on the anisotropic arrangement of nonmagnetic atoms, altermagnetism and AHE may arise in crystals of more than two magnetic sublattices, which can benefit from the same magnetic Laue group analysis.

In spintronic devices, AHE in altermagnets and other AFMs readily enables electrical readout for memory applications while keeping the overall magnetization small. The Hall conductivity can be switched on and off or change sign when the Neel vector is flipped or rotated, or when the material goes through magnetic phase transitions.

### 3.2 Spin Splitter Torque

The spin splitter torque (SST) effect is an analogue of the spin transfer torque effect and converts an electrical current to a spin current. The spin transfer torque arises in FMs and has greatly revolutionized the field of spintronics in the manipulation of magnetization without an external magnetic field: A polarized current from a fixed FM layer can alter the orientation of the magnetization of another FM it flows through by a transfer of angular momentum. This phenomenon can be used to realize magnetic random-access memory (MRAM) in engineered multilayer magnetic heterostructures via out-of-plane currents.^55^ SST, supposedly with similar capability of field-free control of magnetization in adjacent FM layers, on the other hand, is unique to altermagnetism in that the charge current (or the electrical bias) is transverse to the spin current generated.^12^ Unlike the AHE in altermagnets, the presence of SST is robust and does not rely on the direction of the Néel vector.

**Figure 5:**
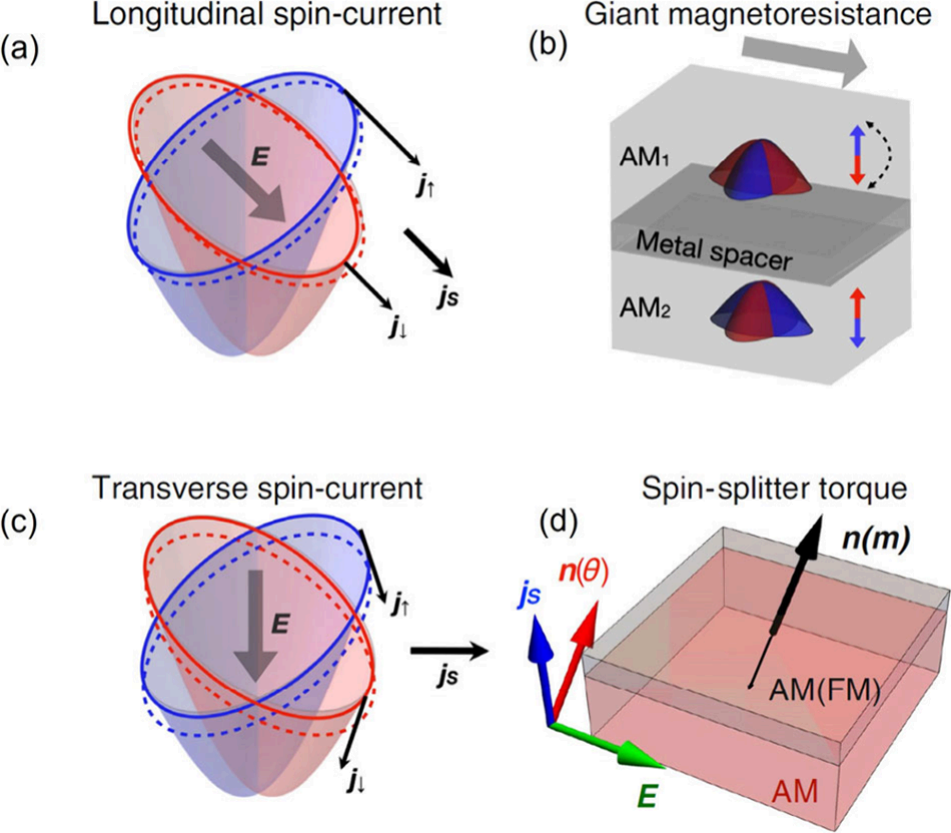
(a) Mechanism of electrical current polarization in *d*-wave altermagnet along a band-splitting direction. (b) Device geometry utilizing two layers of *d*-wave altermagnets separated by a nonmagnetic metal spacer layer with a current-in-plane mode. (c) Mechanism of generating a pure spin current transverse to the external electrical bias (and the electrical current) that is applied along a nodal direction. (d) Schematic of utilizing out-of-plane spin current from the lower *d*-wave altermagnet layer to polarize an FM or Altermagnetic layer above. Figures reproduced from ref (12). Available under a CC-BY 4.0 license. Copyright 2022 Libor Šmejkal.

SST was first theoretically proposed in RuO_2_.^56^ The effect is closely related to the spin polarization effect of *d*-wave altermagnets originated from the anisotropy of spin-dependent Fermi surfaces. In RuO_2_, when a bias is applied along either [110] or [11̅0], the different Fermi velocities of spin-up and down carriers quantified by a gradient normal to the respective Fermi surface leads to a spin-polarized current (Figure 5a), which is highly useful itself. Furthermore, according to theory, when the bias is in between the two polarized directions, along the spin-degenerate direction *x* for example, the group velocities of carriers of both spin types are identical along the field direction but will yield opposite components for opposite spins transverse to the charge current, along *y* in this case (Figure 5c). The latter gives rise to a pure spin current orthogonal to the direction of charge transport or electrical bias. The direction of spin polarization is aligned with the Néel vector. If the material can be grown into a thin film oriented normal to *y* (or *x*), then an in-plane electrical bias along *x* (or *y*) can lead to this out-of-plane spin current to switch the magnetization of a nearby FM layer in the heterostructure (Figure 5d). This anisotropy in spin current generation is crucial for the practicality of SST in devices,^57^ as it allows for tailored magnetization switching depending on the crystal orientation utilized in the device architecture.

It is worth noting that SST is also technically most significant in *d*-wave altermagnets and vanishes as the momentum contrast between the spin-opposite Fermi surface decreases with an increasing number of nodal planes.

### 3.3 Giant and Tunneling Magnetoresistance

Giant magnetoresistance is a phenomenon arising from multilayer FMs with applications for spin valves, field sensors, MRAM, and hard disk drives.^58^ A basic functioning unit contains two FM layers separated by a nonmagnetic layer. Depending on whether the two FM layers have their moments aligned or anti-aligned, the unit will exhibit low or high resistance, respectively, due to the spin-dependent scattering of electrons, which is strongest when the electron spins are antiparallel to the moments and weakest when parallel. The contrast between these two states should ideally be as large as possible to enhance error tolerance and is often significantly larger in multilayer structures than the anisotropic magnetoresistance in a single layer. Giant magnetoresistance can be implemented in two device geometries, i.e., current-in-plane or current-perpendicular-to-plane relative to the FM layers. A current-in-plane schematic is shown in Figure 5b.

The counterpart of giant magnetoresistance in altermagnets originates from the current polarization effect. Therefore, it is only realized in *d*-wave altermagnets. As described in the prototype case of RuO_2_, a spin-polarized current can be generated if the bias is applied along either [110] or [11̅0]. The direction of spin polarization can be reversed when the Néel vector is flipped or if the bias is rotated by 90°.^12^ In a multilayer device made of two altermagnet layers separated by a conductive nonmagnetic interlayer, depending on whether the Néel vectors of two altermagnetic layers are parallel or antiparallel, the stack will exhibit a low or high resistance state, respectively.

If the conductive nonmagnetic layer is replaced by a thin insulator as a barrier, a magnetic tunnel junction is obtained and can be used for measuring tunneling magnetoresistance, which has similar sensing and memory applications as giant magnetoresistance.^59^ In an FM tunnel junction, the relative resistance change of parallel and antiparallel states varies widely from a few to several hundred percent. Likewise, the relative orientation of the momentum-dependent spin-polarized bands of two altermagnetic layers is expected to alter the resistance of the tunnel junction.^12^ The relative change of resistance is expected to reach 100% for RuO_2_ and Mn_5_Si_3_.^60^ Other factors such as the crystal symmetry and electronic structure of the insulating spacer, and their compatibility with the magnetic layers also significantly affect the tunneling magnetoresistance ratio. It is worth noting that unlike giant magnetoresistance, tunneling magnetoresistance can theoretically arise in all altermagnets regardless of the spin-momentum symmetry.

### 3.4 Anomalous Nernst Effect

The Nernst effect refers to the transverse electrical bias generated by a temperature gradient and a magnetic field in a mutually orthogonal configuration, thereby transforming heat to electricity for energy conversion and thermal detectors. The transverse geometry was said to improve conversion efficiency compared to the longitudinal geometry of the analogous Seebeck effect. In FMs, the bias can be generated even in the absence of a magnetic field and is thought to scale up with net magnetization, an effect referred to as the anomalous Nernst effect (ANE).

Recently, ANE was also discovered in a handful of magnetic and nonmagnetic Dirac and Weyl semimetals, such as Cd_3_As_2_,^61^ Co_3_Sn_2_S_2_,^62^ Co_2_MnGa,^63^ in which the nontrivial Berry curvature spikes near the Fermi level in the momentum space in the proximity of Dirac or Weyl points.^61^ This mechanism goes beyond the common association of ANE and net magnetization. Lately, the material space for realizing ANE is further expanded to magnetization-neutral altermagnets. This is because Berry curvature tends to maximize near band anticrossings.^20^ In altermagnets, the crossing nodal lines and planes between spin-up and down Fermi surfaces might be slightly gapped by the spin–orbit coupling, thus giving rise to Berry curvature hotspots and strong ANE.

The rise of ANE relies on the nonvanishing integral of the Berry curvature like the AHE, and thus follows similar symmetrical requirements as outlined above. This thermal–electric analogue to the AHE expands the potential functionality of this emerging class of altermagnetic materials.

## 4 Existing Material Systems

### 4.1 The Rutile Family

**Figure 6:**
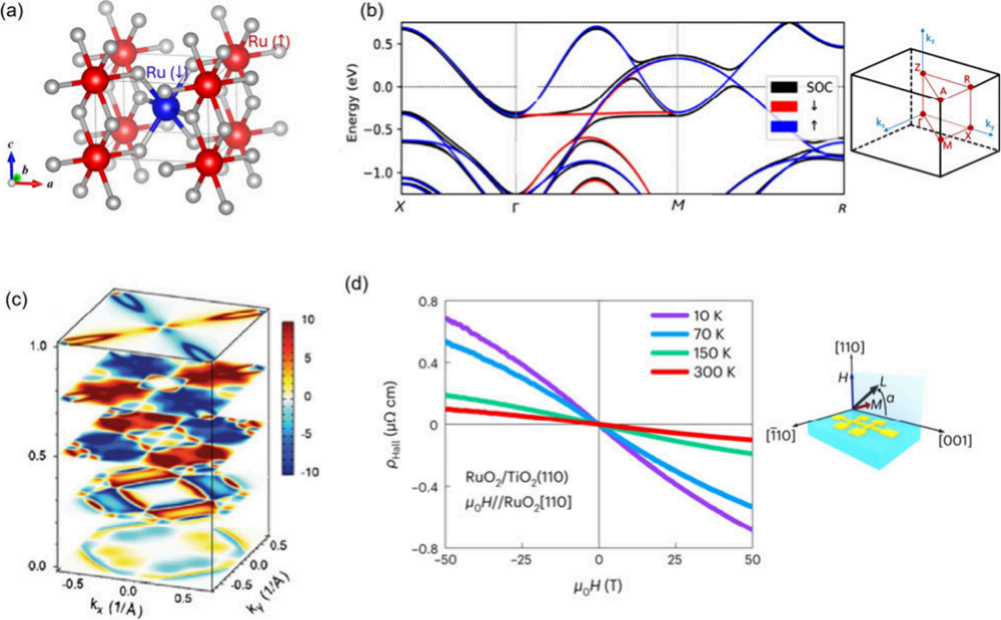
(a) Rutile-type crystal structure. The two opposite-spin sublattices are in blue and red. The coordinating anions are in gray. (b) Electronic band structure calculation of RuO_2_, showing band splitting in blue and red without SOC (Brillouin zone on the right). Calculation with SOC is in black. (c) Simulated Fermi surface for selected ***k***_*z*_ showing ***k***-dependent polarization. (d) Hall resistivity measured on a RuO_2_/TiO_2_ (110) thin film showing nonlinear anomalous contribution. (b-c) reproduced from ref (12). Available under a CC-BY 4.0 license. Copyright 2022 Libor Šmejkal. (d) Reproduced with permission from ref (68). Copyright 2022 Springer Nature.

RuO_2_ is by far the most-studied altermagnet. It adopts a simple rutile-type crystal structure with a collinear antiferromagnetic order (Figure 6a-c) and a *T*_N_ up to at least 300 K.^64^ The material is a rare metallic conductor among rutile-type AFMs due to the less localized Ru 5*d* orbitals. An AHE was predicted via DFT calculations by Šmejkal et al. in 2020.^65^ They demonstrated that the O coordination environment around Ru atoms effectively lifts the *PT* symmetry that is otherwise present in the magnetic spin structure, leading to nearly eV-scale spin splitting comparable to FMs despite zero magnetization. For this reason, the type of AHE in RuO_2_ is also referred to as a crystal Hall effect.

Pure bulk RuO_2_, however, has an inconvenient Néel vector axis along the [001] direction for the moments, as observed through neutron diffraction from single crystals (note that there is still controversy over this identified magnetic structure),^64,66,67^ which leads to a vanishing Hall pseudovector that is excluded by symmetry.^20^ The rationale given is that when moments are in the [001] direction, the magnetically ordered structure retains two 2_1_ screw rotation operations along the [100] and [010] directions, such that a nonzero Hall vector cannot keep invariant under these two orthogonal rotation axes.^20^ When the Néel vector is artificially constrained in the (001) plane, a sizable Hall conductivity is predicted: when the Néel vector is parallel to [100], the Hall vector points in the orthogonal [010] direction; when the Néel vector is in the face-diagonal [110] direction, the corresponding Hall vector is parallel to the Néel vector.

The following experimental pursuit of AHE in RuO_2_ therefore leveraged a prior observation that the Néel vector can be tilted away from [001] by applying an external magnetic field.^65^ In addition, Néel vector rotation was also predicted to be possible through introducing off-stoichiometry or doping. Feng et al. measured Hall responses in deposited RuO_2_ thin films in three orientations, (001), (100), and (110).^68^ While the Hall resistivity has a small and linear field dependence for the (001) and (100) oriented films, which is attributed to an ordinary Hall effect due to a Lorentz force on the moving charge carriers, the Hall resistivity of the (110) film is significantly larger with a nonlinear contribution under the same field, as shown in Figure 6d. The nonlinearity in Hall conductivity, when isolated by subtracting the ordinary Hall component, was estimated to be >1,000 S/cm. It was attributed to AHE due to the tilting of the Néel vector away from [001] (now with a [110] component) to lower the symmetry of the magnetically ordered crystal structure and the subsequent rise of a nonzero Hall vector along [110]. The rotated Néel vector is in between [001] and [110], in the (11̅0) plane, as evidenced by the vector magnetometry measurements.

Also belonging to this structure family are band insulators MnF_2_, CoF_2_, and NiF_2_. These fluorides adopt the same rutile structure and share the same pattern of AFM order as in RuO_2_. The Neel temperatures for MnF_2_, CoF_2_, and NiF_2_ are 67, 37, and 73 K, respectively.^69−72^ MnF_2_ and CoF_2_ have their Neel vectors along the *c* axis while NiF_2_ has an in-plane eas*y*-axis along *a*.^73^ Although their large band gaps exclude possibilities for electrical transport properties, recent theoretical work highlighted possible anomalous thermal Hall effect sensitive to the Neel vector orientation in these insulating systems.^74^

### 4.2 NiAs-Type

**Figure 7:**
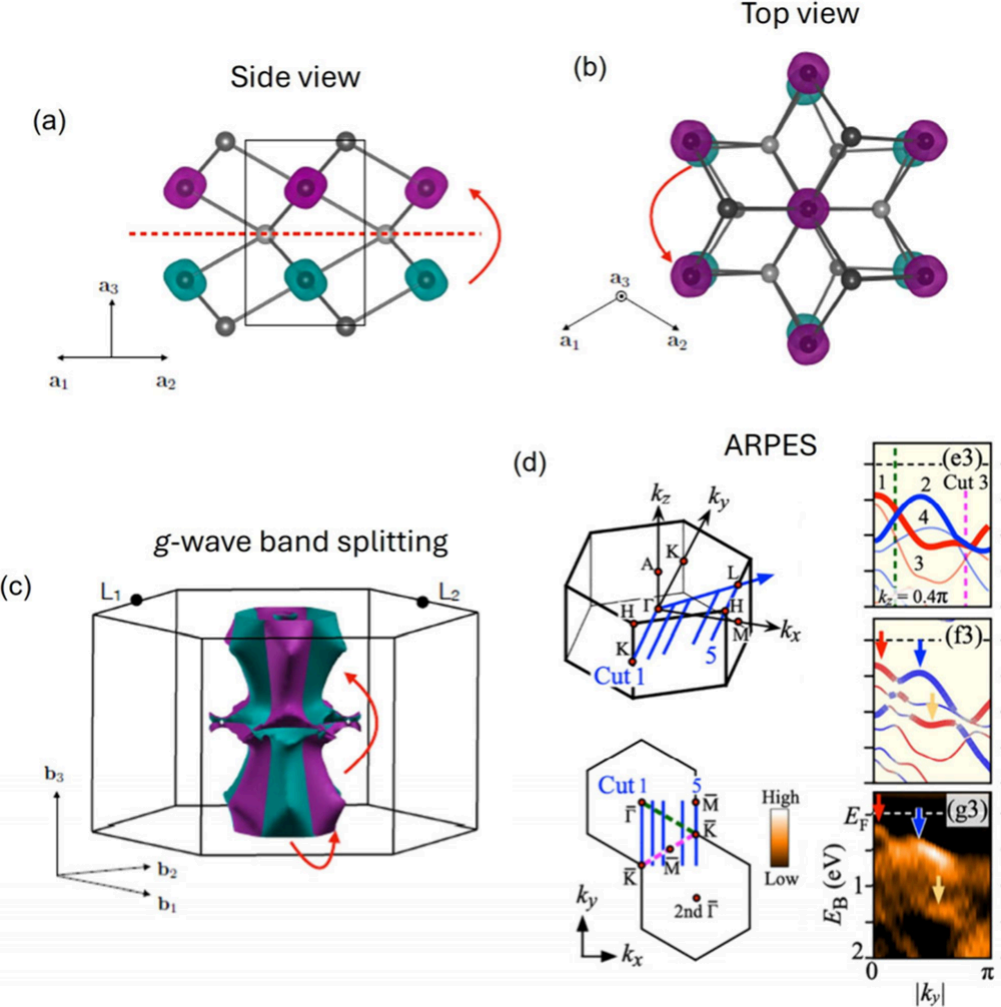
NiAs-type crystal structure in a side (a) or top view (b). The magnetic atoms are highlighted in purple and green to indicate opposite spins. (c) *g*-wave type spin polarization (in green and purple) in the momentum space with the same 2-fold and 6_3_ rotation symmetry between opposite spins as highlighted by red arrows in the real-space structure (a,b). (d) ARPES measurement along Cut 3 (middle blue path shown in the Brillouin zone). On the right, from top to bottom, DFT calculations without and with SOC and ARPES intensity. (a–c) Reproduced from ref (14). Available under a CC-BY 4.0 license. Copyright 2022 Libor Šmejkal. (d) Reproduced with permission from ref (83). Copyright 2024 American Physical Society.

The classic NiAs-type structure is capable of hosting altermagnetism because of the way magnetic atoms are ordered in the hexagonally close-packed anion framework. The magnetic cations adopt a triangular pattern in the plane and stack right on top across layers, forming a locally non-centrosymmetric and trigonal prismatic coordination environment around the anions. The two altermagnets thus far identified in this family, i.e., MnTe and CrSb, both have FM in-plane interaction and AFM interaction between the layers with a (0,0,0) magnetic propagation vector.^75,76^ The opposite-spin sublattices are therefore connected by a 6_3_ rotation along c (Figure 7b) as well as a 2-fold in-plane rotation (Figure 7a). The NiAs structure hosts inversion centers in the cation plane in the atomic structure. Once the cation layer in MnTe or CrSb is FM ordered, the combination of inversion and TRS is lifted. Both materials have a *g*-wave type spin-momentum locking with four non-SOC nodal planes (B-4 type), as shown in Figure 7c.^77^

MnTe has a *T*_N_ slightly above room temperature at ∼307 K. The material is interesting not only for potential spintronic applications but also for its promising thermoelectric properties due to short-range magnetic order developed when doped with alkali metals.^78−81^ The altermagnetic band splitting was observed using temperature-dependent angle-resolved photoemission spectroscopy (ARPES) in comparison to first-principles calculations (Figure 7d) as well as spin-resolved ARPES.^82,83^ Note that spin-resolved ARPES for probing altermagnetic spin splitting is challenging due to the oftentimes coexisting TRS-related domains of reversed moments.^83^ Below *T*_N_, the Mn^2+^ spins are aligned along the [11̅00] direction, lowering the overall symmetry of the magnetic structure to a Laue group of m′m′m. It leads to a nonzero AHE pseudovector along the *c* axis, following the symmetry classification outlined in the AHE section above, which allows the observation of AHE with any in-plane currents. AHE was experimentally observed in a MnTe thin film of an (0001) orientation and is switchable by a field with an out-of-plane component.^21^ It is believed that the small relativistic magnetization along *c* is coupled to the Néel vector to facilitate the switching but is too small to account for the emergence of AHE, which is instead a result of TRS breaking and altermagnetic band splitting. The magneto-transport measurements also revealed that an in-plane field enhances the magnitude of AHE by a spin-flop transition at ∼2 T.

Notably, piezomagnetic effect was observed in MnTe, i.e., mechanical stress induces a measurable magnetization. The piezomagnetic coefficient was measured to be 1.38 × 10^–8^ μB/Mn/MPa at 300 K.^84^ The property not only enables control over the magnetic domains related by TRS, which is typically challenging using traditional magnetic fields due to the absence of a net magnetization, but also serves as a potential diagnostic tool to detect broken TRS. The linear relationship between magnetization and applied stress indicates that even small mechanical stresses can effectively alter the magnetic state of MnTe.

CrSb has a much higher and desirable *T*_N_ at around 700 K. Its same A-type AFM pattern as MnTe renders the same B-4 or *g*-type spin momentum locking (without considering SOC).^85^ Its nearly 0.6 eV band splitting due to altermagnetism was observed immediately below the Fermi level using ARPES.^86^ The magnetic structure as determined by neutron diffraction, however, shows moments aligned along the *c* axis. It contradicts with MnTe in that it adopts a different magnetic point group 6′/m′mm′ that renders a zero pseudo Hall vector and precludes the existence of AHE. A nonlinear dependence of the Hall resistivity on the magnetic field was still observed at low temperatures and tentatively attributed to a multicarrier effect. The magnetoresistance ratio measured of CrSb, defined as the percentage change in resistivity in the presence of a magnetic field, exceeds 30% at 4.2 K and shows no sign of saturation up to 9 T. The multicarrier analysis indicated the presence of carriers with high mobilities, likely due to the presence of Weyl points in the electronic structure.^87^

Note that the in-plane FM order in MnTe and CrSb keeps the 3-fold improper rotation in the same-spin sublattice, and the many nodal planes resulted in the spin momentum locking precludes the possibility of generating polarized current in these materials.

### 4.3 Mn_5_Si_3_

**Figure 8:**
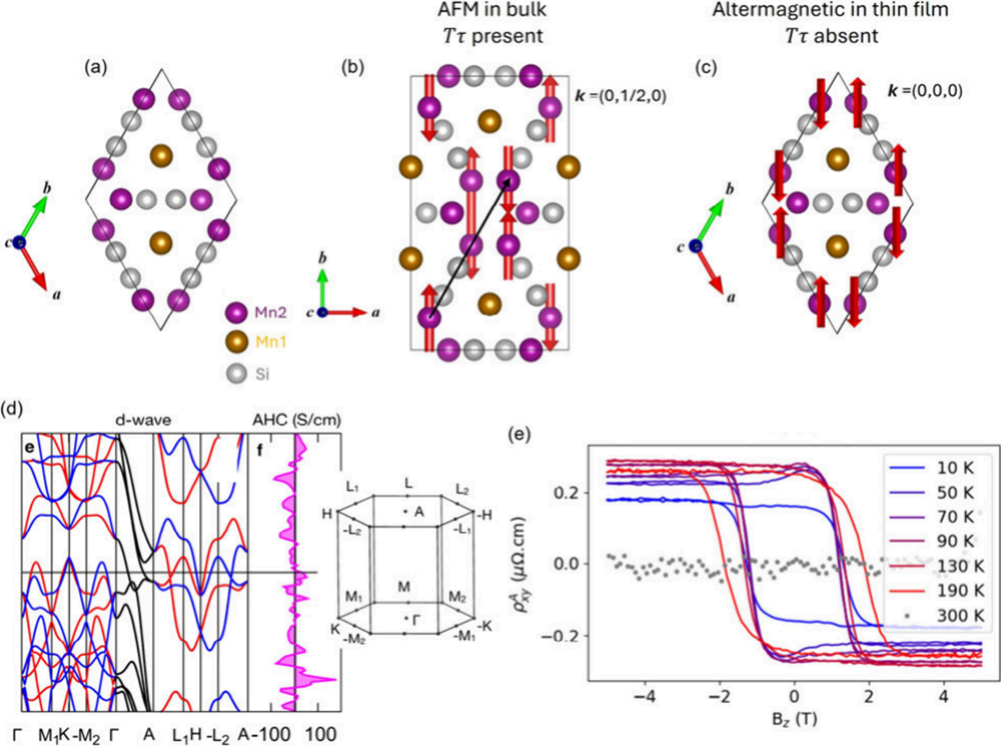
(a) Atomic crystal structure of Mn_5_Si_3_. (b) Bulk AFM magnetic structure below 100 K and above 70 K with a collinear order on 2/3 of the Mn2 atoms. The combination of time-reversal “*T*” and translation “τ” symmetry is present, leading to a trivial AFM state. (c) Thin-film magnetic structure with a different collinear order that lacks “*Tτ*”, leading to an altermagnetic state. (d) Band structure calculation based on the thin-film magnetic order, showing *d*-wave splitting into majority and minority channels and estimated anomalous Hall conductivity (AHC). The Brillouin zone is shown on the right. (e) Anomalous Hall resistivity at various temperatures in a Mn_5_Si_3_ (0001) thin film. (d,e) Reproduced from ref (96). Available under a CC-BY 4.0 license. Copyright 2024 Helena Reichlova.

Mn_5_Si_3_ is a highly unusual intermetallic altermagnetic candidate of its own kind. Both thin films and single crystals grown from Bridgman or flux are available.^88,89^ Above the magnetic transition temperature, the crystal structure adopts a hexagonal symmetry with space group *P*6_3_/*mcm*. The Mn atoms occupy two different Wyckoff positions, i.e., a 4*d* site at (1/3,2/3,0) and a 6*g* site at (0.23,0,1/4), as shown in Figure 8a. A short Mn–Mn bond distance of ∼2.4 Å is observed between the 4*d* Mn sites (Mn1), indicative of metal–metal bonds typical for intermetallics.

The compound exhibits rich magnetic order at low temperatures and considerable contrast between bulk and thin-film samples. In bulk, below ∼100 K and above the second transition temperature between 60 and 70 K, an AFM order develops with collinear moments on 2/3 of the Mn2 (6*g*) and zero-ordered moments on the remaining Mn sites, and the overall structure distorts to an orthorhombic one.^90^ The structural distortion and a magnetic propagation vector (0, 1/2, 0) doubles the unit cell along *b* (Figure 8b) and results in the presence of combined TRS and translational symmetry and renders bulk Mn_5_Si_3_ a trivial AFM in this temperature range. Below ∼70 K, while 1/3 of Mn2 remains nonmagnetic, all Mn1 atoms attain ordered moments. The magnetic structure based on neutron diffraction becomes noncollinear and noncoplanar with a non-centrosymmetric space group of Ccmm,^25^ giving rise to spontaneous AHE due to a topological Hall effect.^91−93^ Applying a magnetic field can restore the collinear magnetic order from the noncoplanar one and the field strength needed increases as the temperature drops.^94,95^

Thin-films samples show subtle yet nontrivial deviation from the bulk. Because of an epitaxial effect, Mn_5_Si_3_ thin films in a (0001) orientation remains the hexagonal symmetry throughout the entire temperature range.^96^ A slightly different collinear AFM order develops with a zero magnetic propagation vector (Figure 8c). Consequently, no transport anomaly was observed to correspond to the ∼100 K transition although the lower temperature anomaly (∼70 K) in the *c* lattice and the longitudinal resistance is preserved. A spontaneous AHE signal was observed (Figure 8e), surprisingly, immediately below ∼240 K. It is believed through comparison with DFT computation that the collinear magnetic structure without the orthorhombic distortion gives rise to *d*-wave type spin splitting (Figure 8d) and the sizable AHE observed.

### 4.4 TM_*x*_NbS_2_ (TM = Co, V, Fe)

Altermagnetism was recently proposed in several TM intercalated Nb or Ta-based dichalcogenides. There is now immense interest in revisiting their magnetic and transport properties. Single crystals of this family are usually obtained through vapor transport growth.^97^

**Figure 9:**
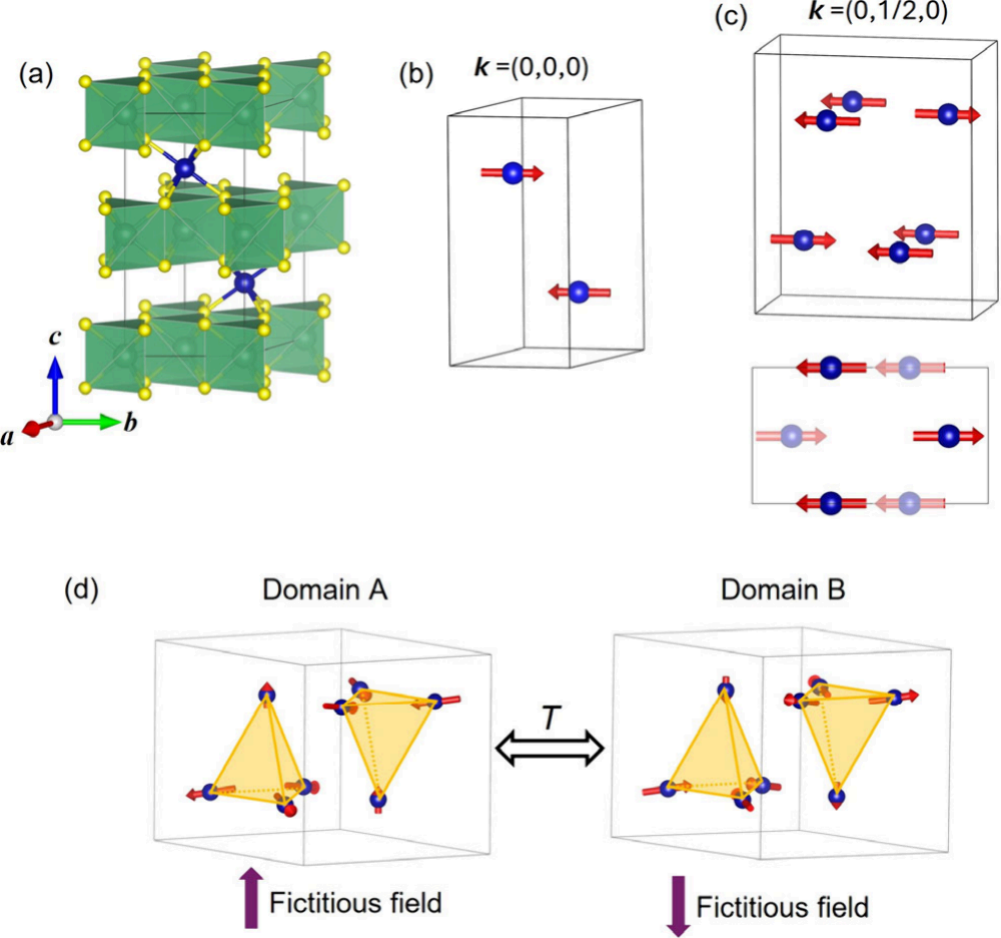
(a) Crystal structure of TM_1/3_NbS_2_, with layers of NbS_6_ trigonal prismatic polyhedra highlighted and blue TM atoms intercalated in between. (b) Collinear magnetic order with zero propagation vector as seen in V_1/3_NbS_2_. (c) Collinear magnetic order with ***k*** = (0,1/2,0) and a supercell, as suggested by a first neutron scattering experiment for Co_1/3_NbS_2_. (d) Noncollinear noncoplanar magnetic order found by single-crystal polarized neutron scattering with degenerate Domains A and B correlated by time-reversal symmetry (*T*).

CoNb_3_S_6_ (or Co_1/3_NbS_2_) is a non van der Waals, layered chalcogenide in which the TM atoms are sandwiched between 2H-NbS_2_ slabs while occupying 1/3 of the available octahedral interstitial sites (Figure 9a). Its structure therefore lacks centro-symmetry due to the trigonal prismatic coordination of Nb and the adopted  Co ordering, resulting in a hexagonal space group *P*6_3_22. The material has garnered significant attention due to its intriguing magnetic properties and large AHE that defies a clear explanation by an existing mechanism such as Dirac or Weyl Fermions, noncollinear or noncoplanar spin textures.^98^ A Curie–Weiss fit at high temperatures found an average moment of 3.0 μ_B_/Co while only a very small intrinsic ferromagnetic component along the *c*-axis at approximately 0.0013 μ_B_/Co was observed below the *T*_N_ of ∼26 K.^99^ An initial nonpolarized neutron study on single crystals at 4.2 K suggested that the material adopts a collinear AFM structure with the moments confined in the *ab* plane (Figure 9c), leading to an orthorhombic magnetic lattice with three equivalent configurational orientations and complex domain structures.^26^ The authors suspected that within each configurational domain, the moments can also be pointing in symmetrically equivalent directions, giving rise to spin domains.

Transport measurements on CoNb_3_S_6_ revealed a critical correlation between the small remanent magnetization and the switchable AHE.^98^ The magnetization flip was seen near *T*_N_ at +1 and −1 T when sweeping a field along *c* between −6 and +6 T immediately below *T*_N_ ∼ 27 K. The coercive field increases, i.e., the magnetization component becomes drastically harder, as the temperature decreases and reaches beyond 6 T once below 24 K. Once the small FM component and its flip are suppressed by higher fields or at lower temperatures, the AHE and its switching behavior vanish. However, the small magnetization alone cannot explain the large AHE observed. The authors suspected that a noncollinear or noncoplanar magnetic structure like in Mn_3_Sn or Mn_3_Ge may be the origin of this behavior.^100^ Band structure calculations with SOC show Weyl nodes near *E*_F_, suggesting a combined result of Weyl nodes and a small magnetization on the electronic structure. A large anomalous Nernst effect was also observed in CoNb_3_S_6_.^101^

The predicted altermagnetism in CoNb_3_S_6_, however, was based on a hypothetical magnetic order wherein the Neel vector is along ***a*** or ***b***, like that of VNb_3_S_6_.^65^ In fact, based on a collinear magnetic structure initially reported (of a nonzero propagation vector and an orthorhombic magnetic unit cell as shown in Figure 9c),^26^ CoNb_3_S_6_ should be a trivial AFM with a type IV magnetic space group. Nevertheless, a recent revisit of the magnetic structure of CoNb_3_S_6_ (and its analogue CoTa_3_S_6_) via polarized neutron scattering on single crystals revealed a noncoplanar arrangement of the magnetic moments below *T*_N_ and a critical field.^102^ Through representation analysis and comparison between the calculated and observed magnetic structure factors, a magnetic unit cell was identified as consisting of 8 Co atoms that form two antiparallel tetrahedra, i.e., one with all-in moments and the other with all-out moments, and breaking the TRS (Figure 9d). This noncoplanar magnetic structure generates a coherent fictitious field experienced by the conduction electrons and contributes to the spontaneous topological Hall effect observed in the material, which does not rely on SOC. Flipping all moments, i.e., applying TRS, will create an equivalent magnetic structure albeit with a reversed fictitious field and opposite Hall conductivity. It is believed that applying an external magnetic field will lift the degeneracy of these two states and the stability of either depends on the sign of the field, which explains the switching behavior of AHE. The work suggests that the non-centrosymmetric crystal structure and the Dzyaloshinskii-Moriya (DM) effect led to small spin canting and the tiny spontaneous magnetization observed before. The tiny magnetization is not a critical component but an add-on effect that accompanies the switching between the two magnetic states.

VNb_3_S_6_ (or V_1/3_NbS_2_) adopts the same crystal structure as CoNb_3_S_6_ and a collinear magnetic order with in-plane moments, ***k*** = (0,0,0) (Figure 9b), although a more recent study revealed more complex out-of-plane AFM order.^27,103^ The absence of centro-symmetry leads to DM interactions and subsequently a small residual magnetization out of the plane. VTa_3_S_6_ behaves similarly to VNb_3_S_6_ except with a lower Néel temperature, i.e., 32 K instead of 50 K.^103^ The combination of TRS and inversion (or translation) is absent in both VNb_3_S_6_ and VTa_3_S_6_, qualifying both materials as altermagnets.

**Figure 10:**
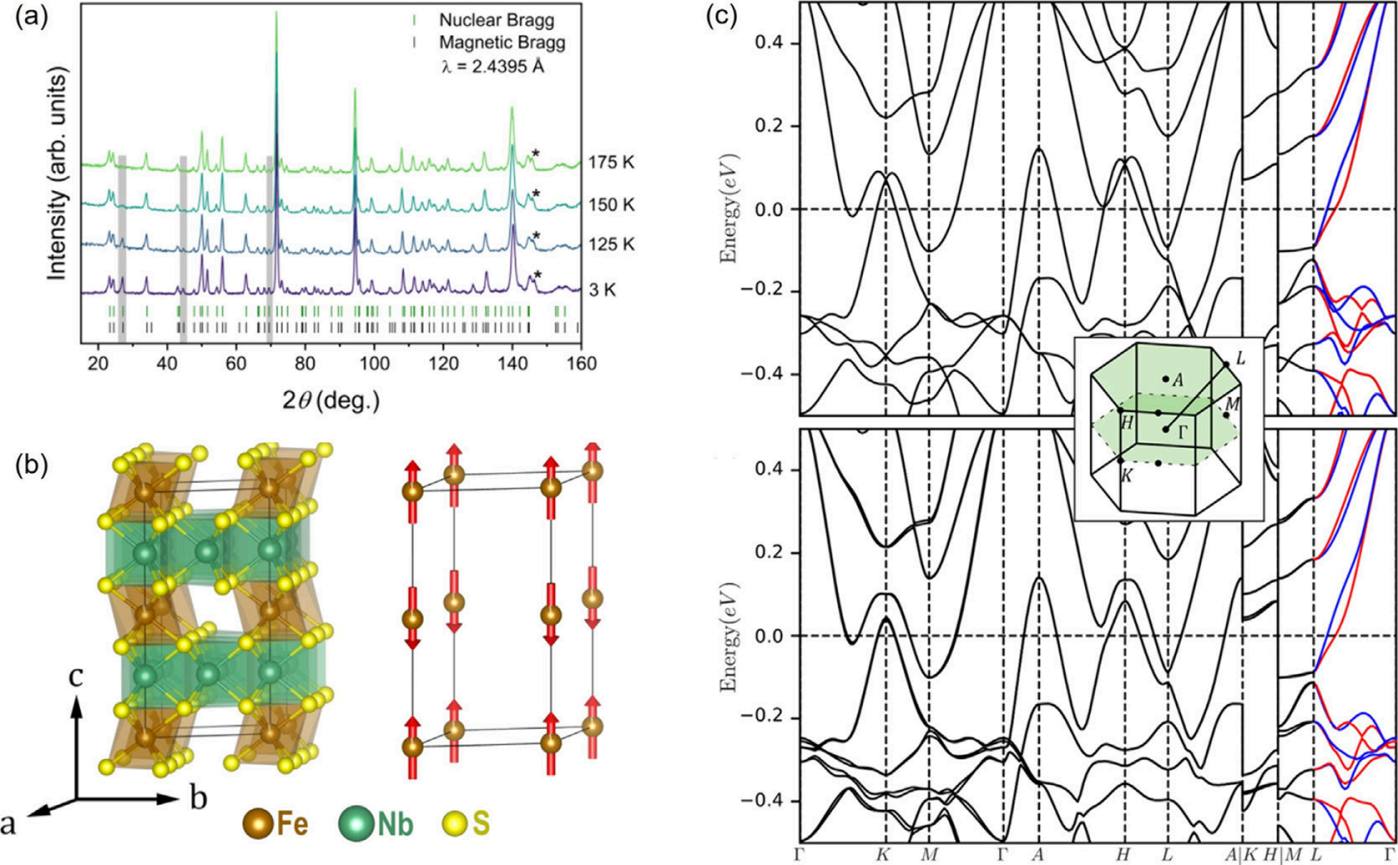
(a) Neutron diffraction of Fe_1/4_NbS_2_ tracking the evolution of diffraction peaks when cooled from 175 to 3 K with the magnetic peaks highlighted in gray. (b) (left) Crystal structure and (right) magnetic structure determined. (c) Spin-projected band structure of Fe_1/4_NbS_2_ (top) with and (bottom) without SOC. Reproduced from ref (104). Copyright 2023 American Chemical Society.

The intercalation of TM in dichalcogenides can also occur at another distinctly lower concentration of *x* = 1/4. While the *x* = 1/3 variants adopt a  TM order and lose centro-symmetry, the *x* = 1/4 ones are centro-symmetric and have a 2*a* × 2*a* TM order. Among these compositions, our group recently identified a new altermagnet, Fe_1/4_NbS_2_.^104^ By *T*-dependent neutron powder diffraction (Figure 10a), its magnetic structure was determined as A-type with in-plane FM coupling and out-of-plane AFM order (Figure 10b). The two opposite-spin sublattices are connected by 6_3_ rotation and time-reversal symmetry, satisfying the criteria of being altermagnetic. Furthermore, due to the absence of horizontal mirror and the presence of a 3-fold rotation axis in the same-spin sublattice point group H, the spin-momentum texture falls in the B-4 type. The spin texture shows four nodal planes, one in the horizontal plane and three vertical ones that are 60° apart. Therefore, among the high-symmetry *k* paths investigated by DFT calculation, only the Γ – *L* path does not fall on a nodal plane and thus exhibits spin polarization in the absence of SOC contribution (Figure 10c). Compared to its non-centrosymmetric Fe-containing analogue Fe_1/3_NbS_2_, which enables electrically switchable memory below *T*_N_ ∼ 40 K following the mechanism (i) outlined in the Introduction,^10^ Fe_1/4_NbS_2_ has a much-enhanced *T*_N_ = 150 K. Nevertheless, its many nodal planes in reciprocal space render it unsuitable for generating spin-polarized conductivity.

## 5 Outlook—A Materials Chemistry Perspective

The discovery of altermagnetism has opened an entirely new materials space for the development of next-generation energy conversion, magnetic sensors and memory devices. Classification based on symmetry analysis has been established for the identification of altermagnetism. Candidates ranging from insulators to metallic conductors can be readily predicted through first-principles DFT calculations. Characterization tools including ARPES and transport measurements for anomalous effects (e.g., AHE, ANE, thermal Hall effect) are increasingly being applied to validate predictions and explore the lesser-known correlations between the complex magnetic ordering and emerging electronic, optical, and thermal properties. From the perspective of materials chemistry, there are ample opportunities for discovering new candidates and better understanding these novel phases.

### 5.1 New Materials Design

The wide chemical space spanning oxides, chalcogenides, pnictides, and intermetallics wherein altermagnetism has been found provides a fertile ground for new candidate discovery. The fact that these new phases and exciting properties do not rely on SOC enables the use of light and earth-abundant elements. Among 3*d* transition metals that often act as magnetic-bearing elements, Cr, Mn, Fe, and Co have the largest spin angular momentums if one assumes that the orbital angular momentum is usually quenched in a solid-state crystal field. Additionally, Mn and Fe are particularly earth-abundant. It is noteworthy that a magnetically compensated state, AFM or altermagnetic, tends to result in or be accompanied by an electrically insulating behavior due to various mechanisms such as Pauli exclusion,^105^ Mott transition,^106^ superexchange or supercell-induced band folding.^107,108^ The materials thus cannot be electrically modified or host electrical transport properties but can still have thermal or optical responses and properties.

### 5.2 Complexity in the Determination of Magnetic Structures

Neutron diffraction is undoubtedly the most powerful and direct probe into the magnetic structure providing that the nuclear crystal structure is already solved from X-ray diffraction or high-temperature neutron diffraction. But it is also noted that an unambiguous solution to the magnetic structure is usually impossible based on powder samples. Single crystals have many advantages over powder for the direct identification of the magnetic propagation vector and the absence of overlap between reflections,^109^ but are harder to obtain. The art of crystal growth, however, is a specialty of materials and solid-state chemists. In the aforementioned example of CoNb_3_S_6_, one realizes that even though a first powder neutron diffraction suggested a collinear AFM ground state, which was the best solution based on a least-squares refinement (though not the only solution), revisiting the magnetic structure determination using polarized neutron scattering on a single crystal is indispensable to explain the exotic AHE properties measured. In light of this example, candidate materials should not necessarily be deemed uninteresting solely as the result of powder neutron experiments. Likewise, a magnetic structure that suggests altermagnetism based on powder samples should be confirmed through single crystal magnetic structure solutions.

### 5.3 Néel Vector Reorientation

The direction of magnetic moments has profound consequences for altermagnets especially for the rise and fall of AHE as well as other anomalous effects. Based on the 10 magnetic Laue groups that allow AHE, the Néel vector should ideally be tilted away from the highest-order rotation axis to allow a nonvanishing Hall pseudovector. For RuO_2_ that contains a 4′ symmetry element, its AHE only appears when the Néel vector is bent considerably away from the “easy” axis (*c*-axis) through an external magnetic field. The ease of reorienting the Néel vector largely depends on the magnetic anisotropy energy (MAE), which is determined by multiple factors such as crystal symmetry, coordination environments, electron configuration, spin–orbit coupling, etc. Other than physical means, chemical substitution will likely affect these factors and therefore alter the direction of the moments. First-principles calculations can predict ground states while neutron diffraction is always needed to verify the magnetic structure.

### 5.4 Contrast between Bulk and Thin-Film Samples

Although bulk polycrystalline and single-crystal samples are ideal for magnetic structure determination using neutron diffraction, thin film will be the ultimate form in device applications. More importantly, by involving substrates and heterostructures in thin-film fabrication, the same material may exhibit crystal and magnetic structures that deviate from bulk. Since the lattice constants and symmetry can be modified through a substrate effect, the magnetic interactions embedded in the atomic lattice might also get affected. Such contrast should be considered in design and strategically leveraged. For example, as a chiral noncollinear AFM, the bulk Mn_3_Sn naturally comes with six degenerate ground-state magnetic states. Remarkably, due to mere 0.2% tensile strain, the number of the degenerate states is reduced to two in thin films to enable bidirectional switching.^110^ Mn_5_Si_3_ (one of the materials we reviewed in this paper) also exhibits distinct crystal and magnetic transitions between bulk and thin films. By suppressing a structural phase transition from hexagonal to orthorhombic in thin films using substrate strain, a nonzero magnetic propagation vector was prevented to allow the emergence of altermagnetism and AHE in the collinear magnetic state.

### 5.5 Atomically Thin van der Waals (vdW) Altermagnets

2D (or atomically thin vdW) materials can also host altermagnetism because even strict plane groups can exhibit rotation symmetries required for the rise of altermagnetism. The realization of altermagnetism does not need crystal exfoliation down to an atomically thin sheet to manifest desired properties. However, a layered structure (and preferably van der Waals gaps for facile cleavage) can be leveraged for easier device making and transport measurements. Furthermore, modifications to physical properties are possible when vdW materials are cleaved down to only a few atomic layers, e.g., the magnetic ordering temperature might be lowered as the interlayer coupling is gradually attenuated in thinner samples;^111^ certain symmetries might be removed when transitioning from 3D to 2D; and the odd- versus even-number of layer-dependent overall magnetization might arise, as seen in MnBi_2_Te_4_.^112^

## Data Availability

The data underlying this study are available in the published article.
